# Design, Optimization, and Modeling of a Hydraulic Soft Robot for Chronic Total Occlusions

**DOI:** 10.3390/biomimetics9030163

**Published:** 2024-03-06

**Authors:** Ling-Wu Meng, Xiao-Liang Xie, Xiao-Hu Zhou, Shi-Qi Liu, Zeng-Guang Hou

**Affiliations:** 1 School of Artificial Intelligence, University of Chinese Academy of Sciences, Beijing 100049, China; menglingwu2019@ia.ac.cn; 2 State Key Laboratory of Multimodal Artificial Intelligence Systems, Institute of Automation, Chinese Academy of Sciences, Beijing 100190, China; xiaoliang.xie@ia.ac.cn (X.-L.X.); xiaohu.zhou@ia.ac.cn (X.-H.Z.); shiqi.liu@ia.ac.cn (S.-Q.L.)

**Keywords:** CTO, soft hydraulic robot, structure optimization, kinematic model, fluid–structure interaction

## Abstract

Chronic total occlusion (CTO) is one of the most severe and sophisticated vascular stenosis because of complete blockage, greater operation difficulty, and lower procedural success rate. This study proposes a hydraulic-driven soft robot imitating the earthworm’s locomotion to assist doctors or operators in actively opening thrombi in coronary or peripheral artery vessels. Firstly, a three-actuator bionic soft robot is developed based on earthworms’ physiological structure. The soft robot’s locomotion gait inspired by the earthworm’s mechanism is designed. Secondly, the influence of structure parameters on actuator deformation, stress, and strain is explored, which can help us determine the soft actuators’ optimal structure parameters. Thirdly, the relationship between hydraulic pressure and actuator deformation is investigated by performing finite element analysis using the bidirectional fluid–structure interaction (FSI) method. The kinematic models of the soft actuators are established to provide a valuable reference for the soft actuators’ motion control.

## 1. Introduction

### 1.1. Research Background

Chronic total occlusion (CTO) is medically defined as a complete blockage in coronary or peripheral arteries with thrombosis in myocardial infarction (TIMI) flow grade 0 of at least three months [1]. The prevalence of CTO in the general population is assumed to be around 15–30% in patients with coronary artery disease (CAD) [2] and 40% in patients with peripheral artery disease (PAD) [3]. More recently, the percutaneous coronary intervention (PCI) operation has been the most popular approach to treating vascular stenosis lesions because of its lower cost, shorter hospitalization stays, and reduced procedural morbidity. Doctors explore many PCI treatment guidelines and operating procedures.

However, compared with other vascular stenosis lesions, CTO is one of the most severe and complicated lesions in the coronary and peripheral blood vessels, with more sophisticated treatment, lower procedural success rate, and more restrictions on therapeutic methods and operation instruments. The most considerable difficulty in treating CTO is that the vessels are entirely blocked by the plaque or thrombus and might even be accompanied by calcification lesions, leading to a barrier to passage of the guidewire. It makes the operation hard to complete.

### 1.2. Related Works

Many scholars and medical companies have proposed and released various dedicated operation instruments or devices for CTO [4] based on different working principles and clinical demands to improve the procedural success rate. This subsection introduces several typical instruments or devices and their treatment strategies for CTO. The Frontrunner XP catheter is a CTO crossing device whose essential part is a blunt microdissection catheter [5,6,7]. It rebuilds a channel for the guidewire’s passage using the distal tip of the catheter. Some scholars have verified its safety and efficacy [8]. The Crosser system utilizes high-frequency mechanical vibrations [9]. Optical coherence tomography (OCT) applies infrared light to assist guidewires crossing through the occlusion, such as the Ocelot system [10]. Other devices include the Wildcat catheter [11], the Viance catheter [12], and the TruePath crossing system [13]. However, these instruments or devices require a guidewire first to pass through the plaque or thrombus. It is difficult for a guidewire to pass through the CTO lesion by itself. Therefore, we must develop a medical device or robot to actively open a channel inside the atherosclerotic plaque that blocks the vessel entirely.

Soft robots have developed from redundant robots to continuum robots to soft robots made of fully soft materials [14,15]. The soft robot in this article is made of fully soft materials. Compared with traditional rigid robots, soft robots have greater potential in the medical field [16,17,18]. The core component of a soft robot is a soft actuator with enough flexibility, output force, and travel capacity. There are numerous published literature studies on soft actuators or soft robots to replicate the locomotion mechanisms of earthworms [19,20,21,22,23,24]. Inspired by the locomotion of earthworms, a novel bionic soft robotic system is proposed. The soft robot can enter the blood vessel and open a channel inside the atherosclerotic plaque or thrombosis by peristaltic locomotion. The guidewire can pass through the channel to arrive at the distal end of the CTO lesion, making the subsequent operation procedures feasible.

### 1.3. Challenges and Contributions

There are some challenges in this work. Firstly, the two main design challenges are the size limitation caused by the application scenarios and the fabrication difficulty caused by the unique material properties. Secondly, the theoretical method of establishing the kinematic model is challenging due to the materials’ hyperelasticity and nonlinearity. The finite element method facilitates building an accurate kinematic model more effectively by numerically characterizing the effect of hydraulic pressure on actuator deformation.

The main contributions of this paper are as follows: (1) A novel active opening device based on the bionic method is developed, representing an innovative improvement to existing passive devices or instruments. Compared with other devices, the most significant advantage or contribution is solving the challenging problem that the guidewire cannot cross through the CTO lesion. (2) Some indicators are proposed to quantitatively evaluate the performance of soft actuators with different structure parameters. (3) Three nonlinear relationships are established among actuator deformation, maximum stress, maximum strain, and hydraulic pressure. The relationship between deformation and hydraulic pressure is applied to describe the kinematic model.

The rest of this paper is organized as follows: Section 2 describes the earthworm’s locomotion principle and the soft robot’s design details. Section 3 investigates the rubber’s constitutive model and the soft robot’s optimal structure parameters. Section 4 shows and discusses the simulation results, which provide a valuable reference for the kinematic model of the actuators. Finally, conclusions are presented in Section 5.

## 2. Bionic Design of the Soft Hydraulic Robot Based on the Earthworm’s Locomotion

### 2.1. Locomotion Principles of the Earthworm

**Figure 1:**
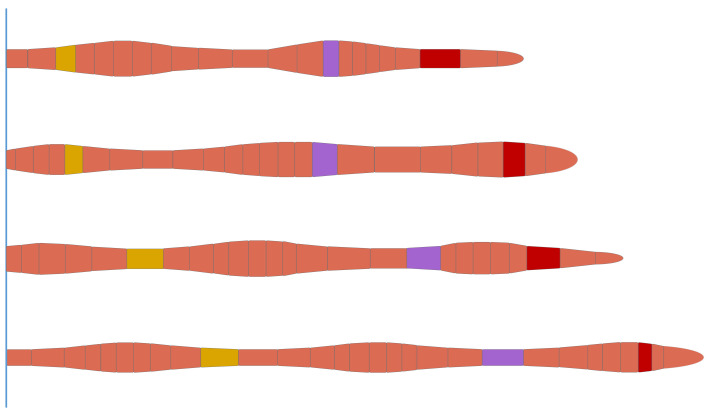
The peristaltic process of earthworms.

**Figure 2:**
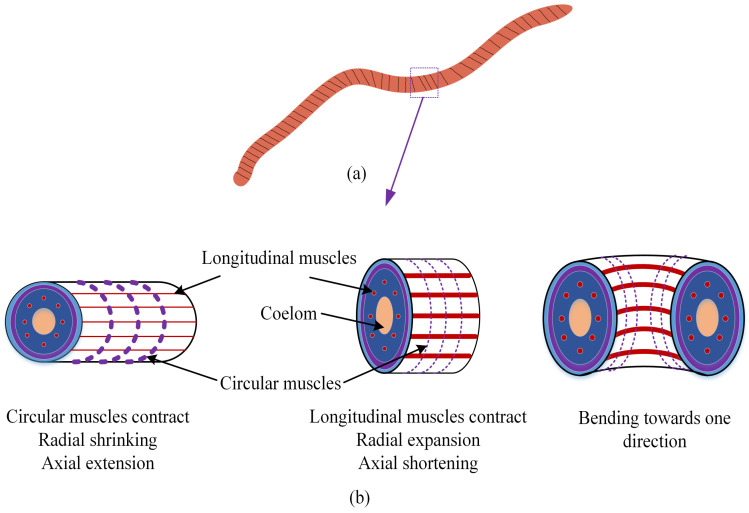
The locomotion principle of earthworms. (**a**) A segment of an earthworm’s body. (**b**) The contraction and relaxation behaviors of muscles.

An earthworm’s body comprises the skin, circular muscles, longitudinal muscles, coelomic fluid, the nervous system, and other parts. As shown in Figure 1, the locomotion of earthworms mainly depends on the body peristaltic waves generated by the contraction and relaxation behaviors of each segment’s circular and longitudinal muscles. This locomotion mechanism enables stability and efficiency in highly unstructured environments like the vessels in this study. The circular muscles contract or relax radially, and the longitudinal muscles contract or relax linearly. The closed coelom made of muscles is filled with fluid, forming a typical hydroskeletal structure. This structure results in an antagonistic relationship between the circular and longitudinal movements. Figure 2 shows a segment of an earthworm’s body. As shown in Figure 2b, when the circular muscle contracts, the earthworm shrinks along the radial direction and extends along the axial direction; when the longitudinal muscle contracts, the earthworm expands along the radial direction and shortens along the axial direction.

### 2.2. Bionic Design of the Soft Actuators

**Figure 3:**
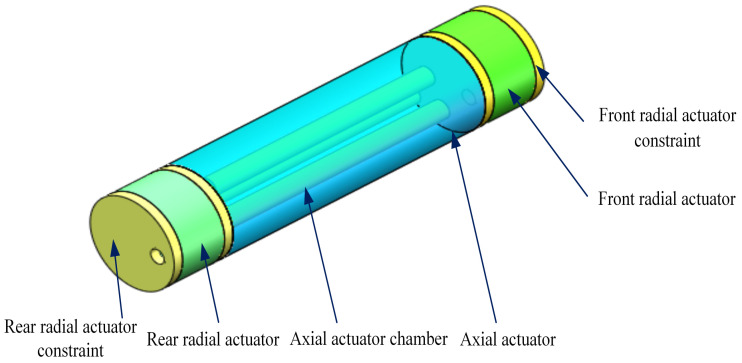
The structure of the three-actuator soft robot.

Figure 3 shows the structure of the biologically inspired soft robot. The soft robot is designed as a three-actuator structure composed of a front radial actuator, a central axial actuator, and a rear radial actuator. The two radial actuators are used to mimic the circular muscles, and the axial actuator is used to mimic the longitudinal muscles. The two radial actuator constraints limit radial actuator deformation along the axial direction.

The three motions to be realized by the soft robot are the expansion of the radial actuator, extension, and the bending of the axial actuator. The hydraulic pressure controls the actuator deformation, which is adjusted by pumping liquid into and out of the actuator chamber. The corresponding relationship between the earthworm’s locomotion and actuator actions is shown in Table 1.

### 2.3. Locomotion Realization

**Figure 4:**
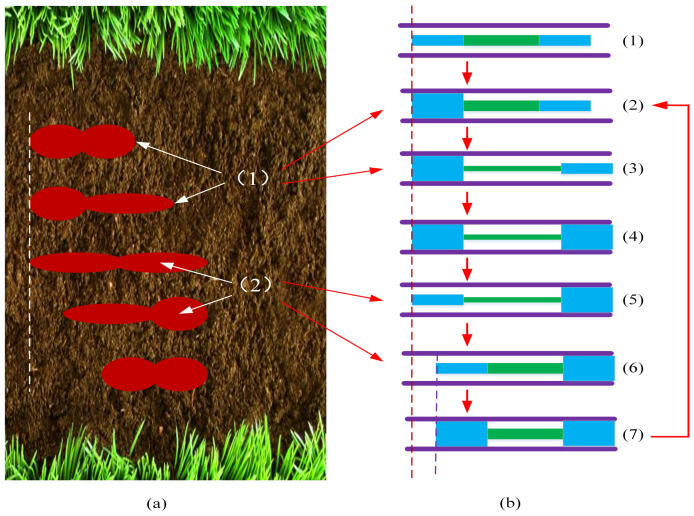
The comparison between the locomotion realization progress of the natural earthworm and the artificial soft robot. (**a**) Locomotion realization progress of the natural earthworm. (**b**) Locomotion realization progress of the artificial soft robot.

Figure 4a shows the locomotion of an earthworm with two body segments. (1) The circular muscles contract, and the earthworm’s head moves forward. (2) The longitudinal muscles contract, and the front segment anchors the ground. Step 1 in Figure 4a corresponds to steps 2 and 3 in Figure 4b. Step 2 in Figure 4a corresponds to steps 5 and 6 in Figure 4b. The steps do not correspond to one another because three actuators are used to mimic the behaviors of the circular and longitudinal muscles.

Inspired by the locomotion of earthworms, we designed the whole locomotion gait according to the actual requirements of treating CTO, as shown in Figure 4b, including the following: (1) Arriving at the lesion site. (2) The rear radial actuator expands. (3) The axial actuator extends. (4) The front radial actuator expands. (5) The rear radial actuator contracts. (6) The axial actuator contracts. (7) The rear radial actuator expands. (8) The front radial actuator contracts; return to step 2. The soft robot moves forward by these cyclic motions, crossing through the occlusion and rebuilding a channel.

### 2.4. Actuation Methods

Scholars have proposed and designed various actuation methods for soft robots or actuators, such as the soft fluidic actuator (SFA) [25], the cable-driven actuator [26], the shape memory alloy (SMA) actuator [27,28], and the electroactive polymer (EAP) actuator [29]. In this paper, we adopt the hydraulic-driven actuator, and for safety, we use normal saline as the driving liquid.

### 2.5. Structure Details of the Soft Actuators

**Figure 5:**
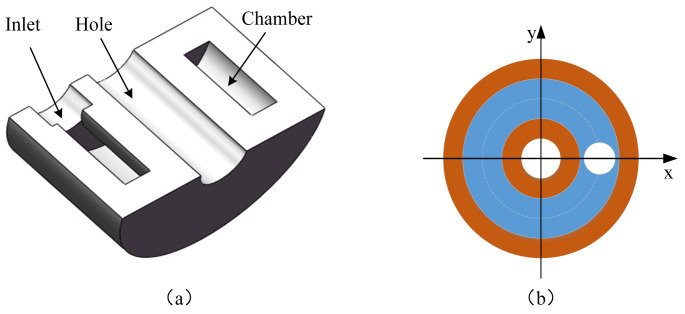
Structure of the radial actuator. (**a**) Cross-section view of the 3D model. (**b**) Inlet location.

The structure of the radial actuator is shown in Figure 5. The diameter and height of the inlet are 1.6 mm and 1 mm, respectively. The diameter and height of the hole are 2 mm and 5 mm, respectively. The diameter and height of the chamber are 1 mm and 3 mm, respectively. We can control the expansion of the radial actuator by adjusting the hydraulic pressure. The expanded size should be slightly bigger than the internal diameter of the vascular lesion site.

**Figure 6:**
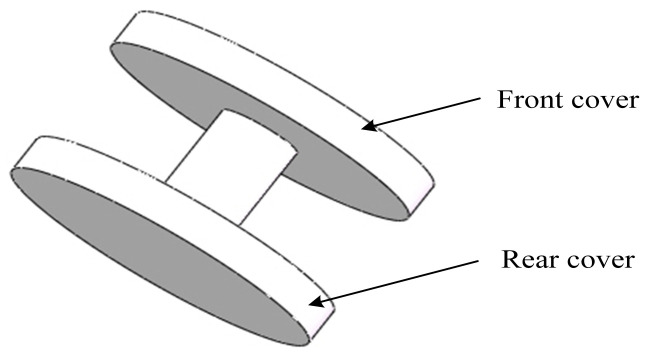
Structure of the axial constraint.

An axial constraint can prevent the radial actuator’s expansion along the axial direction. Figure 6 shows its structure. The diameter and height of the front cover are 8 mm and 1 mm, respectively.

**Figure 7:**
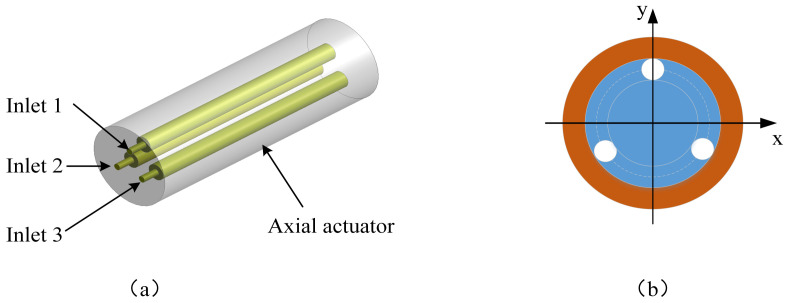
Structure of the axial actuator. (**a**) Perspective view. (**b**) Inlet configuration.

The structure of the axial actuator is shown in Figure 7. The axial actuator is designed as a three-chamber structure to realize the expansion or bending motion by the difference and changes among the hydraulic pressures of the axial actuator’s three chambers. The axial actuator expands along the axial direction when the three chambers’ hydraulic pressure values are the same. When the three chambers’ hydraulic pressure values differ, the axial actuator bends in different directions. The PE wire is wound on the surface of the axial actuator to prevent radial expansion. The diameter and height of the axial actuator are 8 mm and 30 mm, respectively. The diameter and height of the chamber are 2 mm and 26 mm, respectively. The diameter and height of the inlet are 1.6 mm and 1 mm, respectively. All three inlets are the same size.

### 2.6. Opening Process

**Figure 8:**
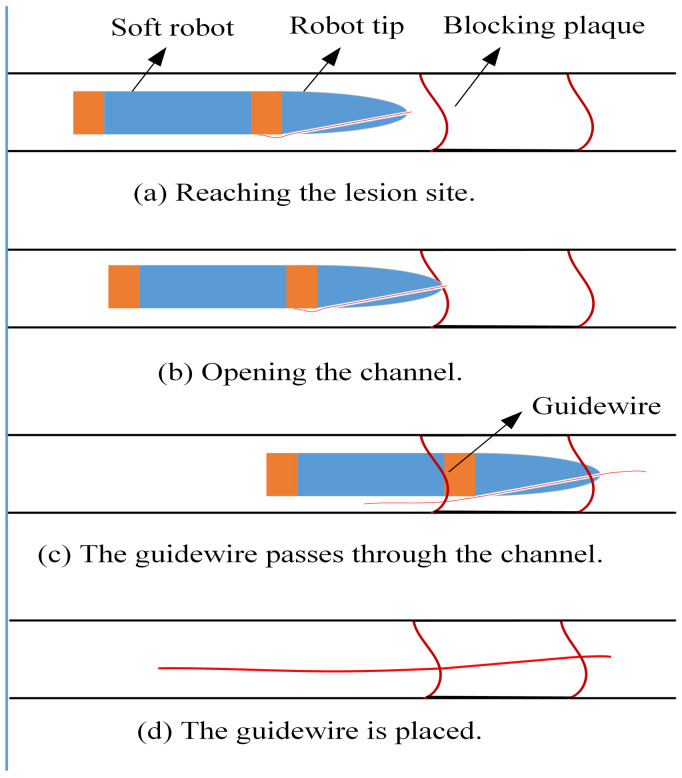
The opening process of the soft robot.

A mechanism based on the tip shape of the front end of the soft robot is designed for the robot to open the plaque actively. Figure 8 shows the progress of using the soft robot to treat the CTO lesion. The first step is to utilize the soft robot and the tip to open the channel actively, and the soft actuators provide power. Secondly, the guidewire passes through plaque via the hollow channel of the tip. Thirdly, doctors perform the standard PCI procedure after the guidewire reaches the distal end of the CTO lesion. The soft robot can realize an active forward or turning motion through these steps, enhancing flexibility and mobility. It can improve work efficiency and operation success rate, with solid clinical practicability and broad potential.

## 3. Structure Optimization

### 3.1. Constitutive Model of the Rubber

Elastic modulus and Poisson’s ratio are inappropriate in the deformation analysis of rubber by the finite element method due to the material’s hyperelasticity and nonlinearity. So, many scholars have proposed all kinds of constitutive models to analyze the mechanical performance of rubber or other nonlinear materials. The Yeoh hyperelastic material model based on the strain energy density function is selected for the finite element analysis in this study [30].

(1) $$W=\sum_{i=1}^{N}C_{i}{\left(I_{1}-3\right)}^{i}+\sum_{k=1}^{N}\left(1/D_{k}\right){\left(J-1\right)}^{2k}$$

where $I_{1}$ is the first deviatoric strain invariant; *N*, $C_{i}$, and $D_{k}$ are material constants; *J* is the ratio of the volume after deformation to that before deformation. Rubber, in this study, is regarded as an incompressible material. For incompressible materials, *J* = 1. The Yeoh model is a function of $I_{1}$ only. So, the binomial parameter form is as follows:

(2) $$W=C_{1}\left(I_{1}-3\right)+C_{2}{\left(I_{1}-3\right)}^{2}$$

### 3.2. Structure Parameter Optimization of the Axial Actuator

**Figure 9:**
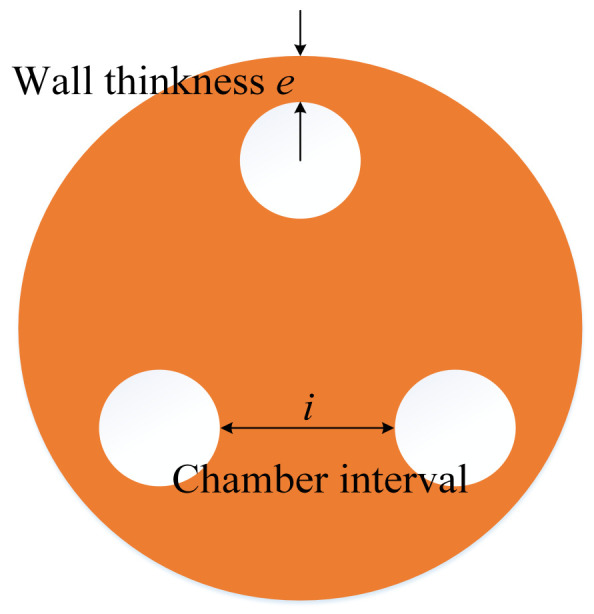
Parameters of the axial actuator.

For the axial actuator, we should analyze the extension and bending motions. The wall thickness (*e*) and the chamber interval (*i*) are the critical structure parameters of the axial actuator, as shown in Figure 9.

#### 3.2.1. Wall Thickness (*e*)

**Figure 10:**
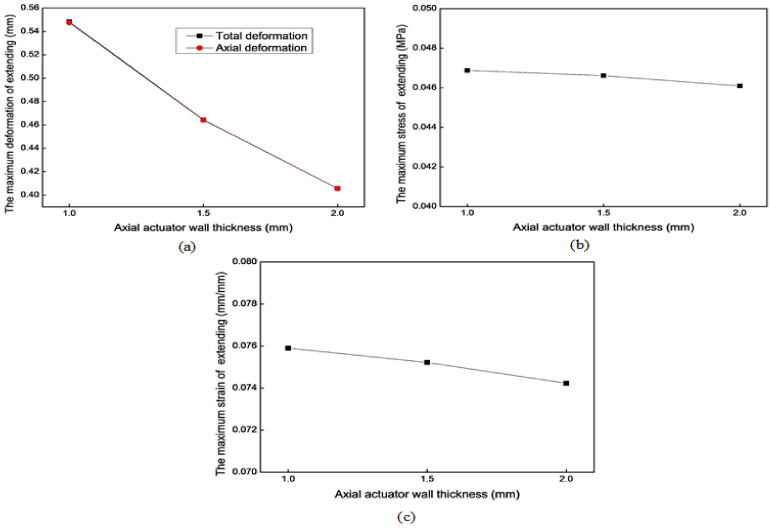
Mechanical results of the axial actuator’s extension motion for different wall thicknesses. (**a**) The maximum total deformation. (**b**) The maximum stress. (**c**) The maximum strain.

Figure 10 shows the mechanical simulation results of the axial actuator’s extension motion for different wall thicknesses. The wall thicknesses (*e*) are 1.0 mm, 1.5 mm, and 2.0 mm, respectively. For the extension motion, the larger the *e* is, the smaller the maximum deformation is. However, *e* does not obviously affect the maximum stress and strain.

**Figure 11:**
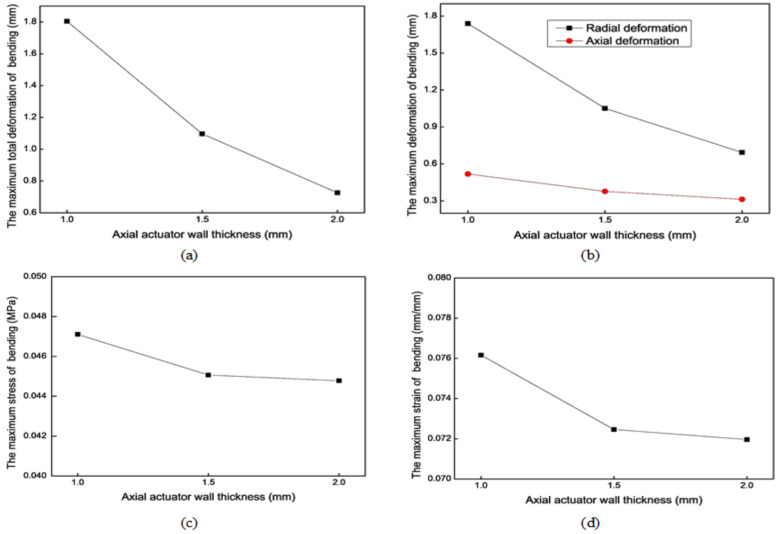
Mechanical results of the axial actuator’s bending motion for different wall thicknesses. (**a**) The maximum total deformation. (**b**) The maximum axial and radial deformation. (**c**) The maximum stress. (**d**) The maximum strain.

Figure 11 shows the mechanical simulation results of the axial actuator’s bending motion for different wall thicknesses. For the bending motion, the larger *e* is, the smaller the maximum deformation is. The effect along the radial direction is more evident than that along the axial direction. However, *e* has no apparent effect on the maximum stress and strain. Considering the extension and bending motion results, the optimal wall thickness (*e*) is 1 mm.

#### 3.2.2. Chamber Interval (*i*)

**Figure 12:**
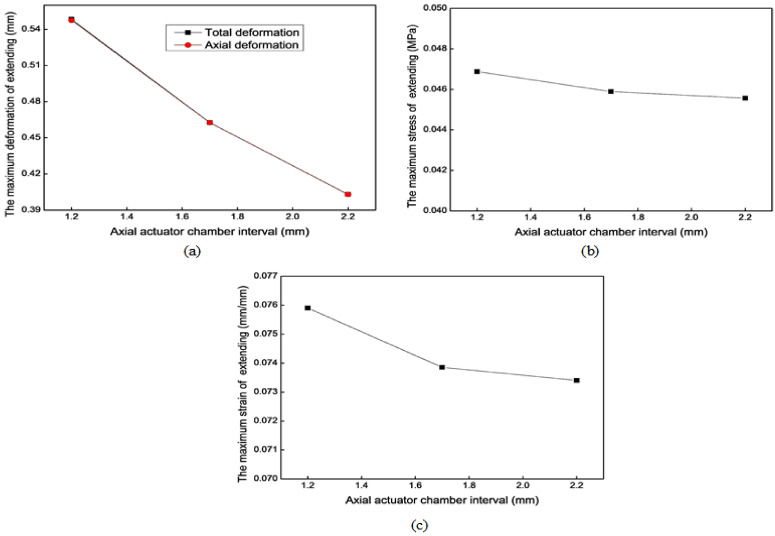
Mechanical results of the axial actuator’s extension motion for different chamber intervals. (**a**) The maximum total deformation. (**b**) The maximum stress. (**c**) The maximum strain.

Figure 12 shows the mechanical simulation results of the axial actuator’s extension motion for different chamber intervals. For the extension motion, the larger the chamber interval (*i*) is, the smaller the maximum deformation is. The effect along the radial direction is more evident than that along the axial direction. However, *i* has no noticeable impact on the maximum stress and strain.

**Figure 13:**
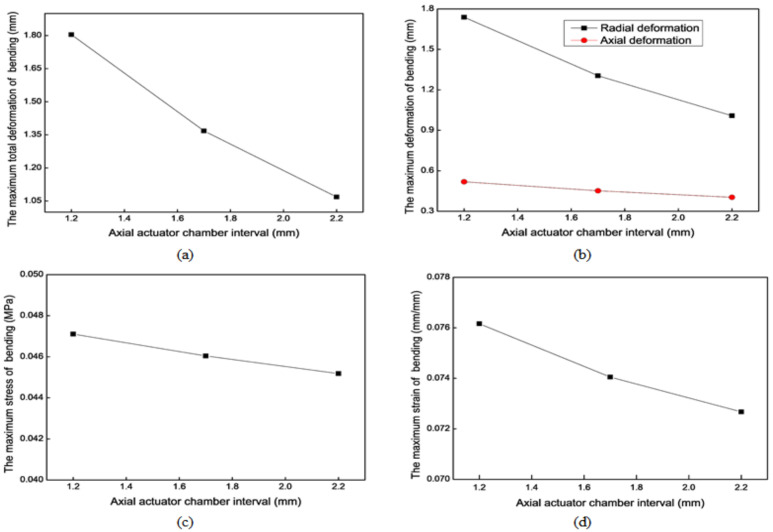
Mechanical results of the axial actuator’s bending motion for different chamber intervals. (**a**) The maximum total deformation. (**b**) The maximum axial and radial deformation. (**c**) The maximum stress. (**d**) The maximum strain.

Figure 13 shows the mechanical simulation results of the axial actuator’s bending motion for different chamber intervals. For the bending motion, the larger *i* is, the smaller the maximum deformation is. The effect along the radial direction is more evident than that along the axial direction. However, the chamber interval (*i*) has no apparent impact on the maximum stress and strain. The extension and bending motion results show that the optimal chamber interval (*i*) is 1.2 mm.

#### 3.2.3. Material Properties

**Figure 14:**
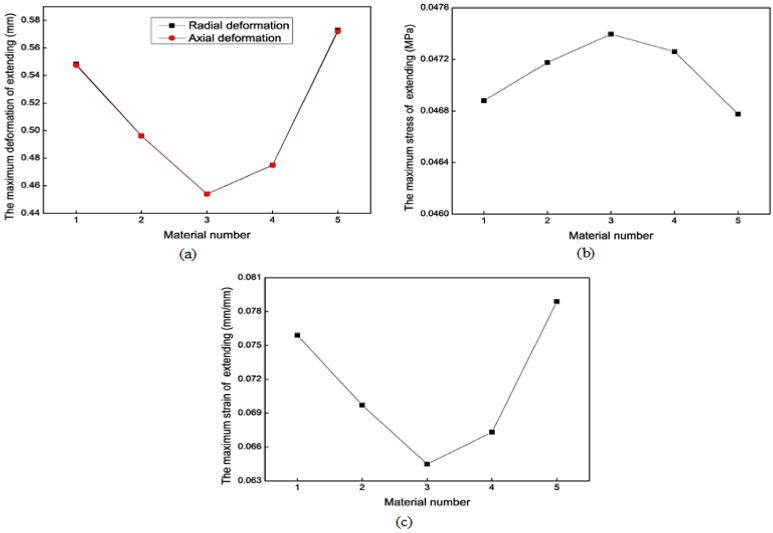
Mechanical results of the axial actuator’s extension motion with different materials. (**a**) The maximum total deformation. (**b**) The maximum stress. (**c**) The maximum strain.

**Figure 15:**
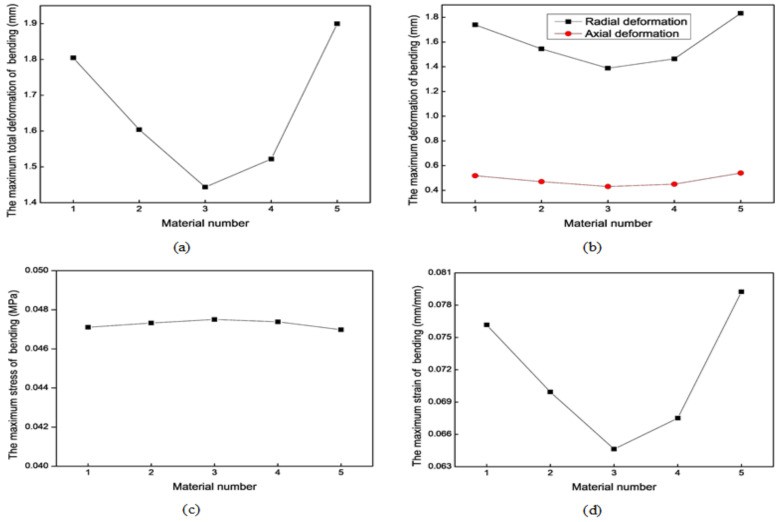
Mechanical results of the axial actuator’s bending motion with different materials. (**a**) The maximum total deformation. (**b**) The maximum axial and radial deformation. (**c**) The maximum stress. (**d**) The maximum strain.

We need to consider the production of soft robots when selecting materials. Soft robots, especially those made of rubber, mainly use Smooth-On company’s materials. We searched for the properties of the materials provided by this company, referring to a large number of literature in the field [31] and making extensive comparisons between the various materials mentioned in the literature. Finally, five materials were chosen. The parameter values of these different materials are listed in Table 2. The simulation results of the axial actuator extension and bending motions with different materials are shown in Figure 14 and Figure 15, respectively, providing some references for choosing the rubber material. Parameter $C_{1}$ substantially affects the results more than parameter $C_{2}$. According to the extension and bending motion results, material No. 1 is optimal. The soft actuators in this study are made of the Ecoflex 00-50 rubber material produced by Smooth-On company, Macungie, Pennsylvania, USA. It is a highly flexible and extensible material with good biocompatibility. The good tensile strength and up to 980% elongation ensure the actuators can withstand larger expansion or extension.

### 3.3. Structure Parameter Optimization for the Radial Actuator

**Figure 16:**
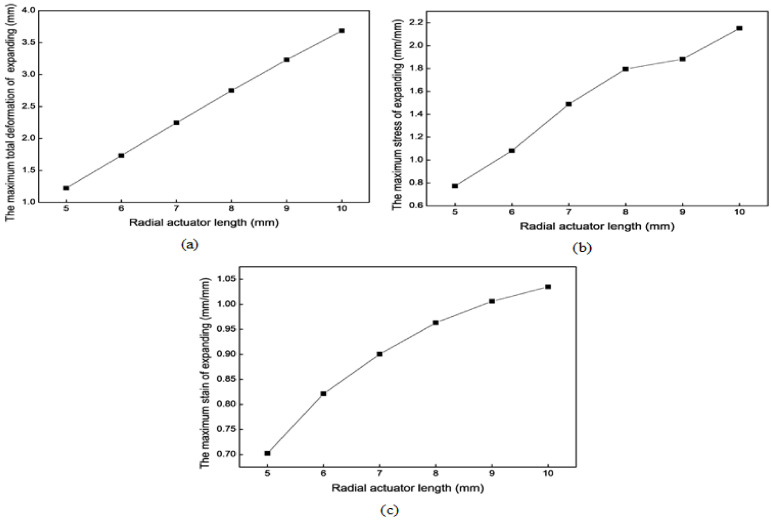
Mechanical results of the radial actuator’s expansion motion for different lengths. (**a**) The maximum total deformation. (**b**) The maximum stress. (**c**) The maximum strain.

The critical structure parameter of the radial actuator is the actuator length. The hydraulic pressure is assumed to be 0.1 MPa in this simulation. Figure 16 shows the mechanical simulation results of the radial actuator’s expansion motion for different actuator lengths. According to the simulation results, the longer the actuator is, the greater the maximum total deformation, the maximum stress, and the maximum strain. Finally, the optimal actuator length is 8 mm.

## 4. Kinematic Models of the Soft Actuators

### 4.1. Selection of the Modeling Methods

The Denavit–Hartenberg (D-H) method is widely used to build rigid robot kinematic or dynamic models. However, there are no systematic theories or methods for soft robot kinematic models. It is difficult to figure out an accurate analytical solution for a hydraulic soft actuator with material hyperelasticity and nonlinearity. The soft robot’s motion control and trajectory prediction become impossible. Domestic and foreign scholars have proposed various valuable methods to establish kinematic models [32,33], including the piecewise constant curvature method (PCC) [34]. We use the FSI simulation method to establish the kinematic model in this study. This method fits the actual working conditions more than static finite element analysis.

### 4.2. FSI Simulation Settings

Hydraulic changes resulting from fluid injection or return exert pressure on the actuator, causing it to deform. The complete FSI analysis performed on the Workbench platform of ANSYS software includes the fluid dynamics simulation based on the Fluent module and the mechanical simulation based on the Mechanical module. The fluid dynamics data in the Fluent module are transferred to the Mechanical simulation module, and the data of the Mechanical module are returned to the Fluent module. The fluid dynamics simulation indicator is the maximum velocity of normal saline water in the chambers. The mechanical simulation indicators are the maximum total deformation, the maximum radial deformation, the maximum axial deformation, the maximum stress, and the maximum strain. These analysis results are fundamental to realizing the soft actuators’ motion control. A mesh independence check is implemented before this simulation. The element number is about 30,000, and the node number is 130,000 to 220,000. The model parameters are set as follows [35]: $C_{1}$ = 0.10 MPa and $C_{2}$ = 0.02 MPa.

### 4.3. Simulation Results of the Axial Actuator

#### 4.3.1. Flow Field

**Figure 17:**
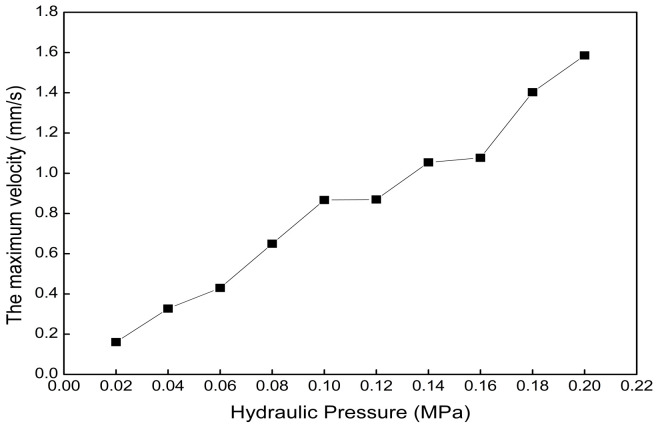
The maximum velocity of normal saline water in the axial actuator chamber under different hydraulic pressures.

The boundary conditions of the fluid dynamics simulation using Fluent software are as follows: steady, laminar, and pressure inlet. The analysis time of the mechanical analysis is set to 1 s. The hydraulic pressure range is 0.02 MPa to 0.2 MPa, with intervals of 0.02 MPa. Figure 17 shows that the maximum velocity of normal saline water in the axial actuator chamber increases with the increase in hydraulic pressure. The relationship between the maximum velocity and hydraulic pressure can be established.

#### 4.3.2. Axial Extension Results

**Figure 18:**
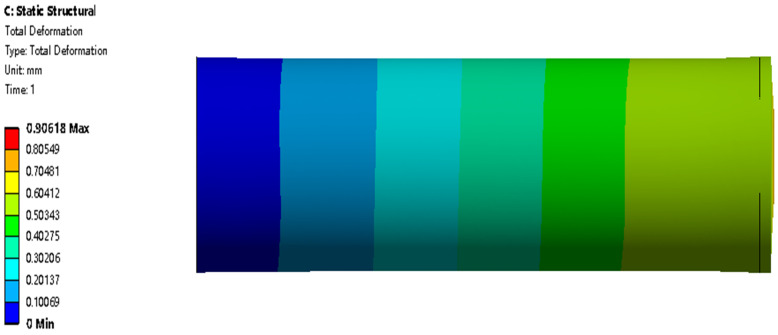
Total deformation contour of the axial actuator’s extension motion when the three chambers’ hydraulic pressures are all 0.1 MPa.

**Figure 19:**
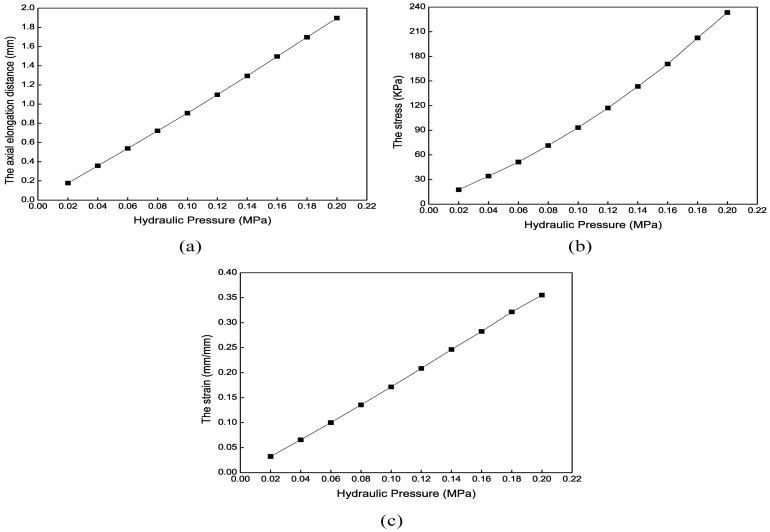
Mechanical results of the axial actuator’s extension motion under different hydraulic pressures. (**a**) The maximum total deformation. (**b**) The maximum stress. (**c**) The maximum strain.

When the hydraulic pressures in the three chambers are the same, the axial actuator is extended or shortened along the axial direction. The actuator extends when normal saline water is pumped into the chambers and shortens when saline is pumped out of the chambers. The maximum total deformation is 0.91 mm in Figure 18. Figure 19 shows that the maximum stress and strain increase with the increase in hydraulic pressure. The maximum total deformations under the different hydraulic pressures provide a reference for controlling the extension distance.

#### 4.3.3. Bending Results

**Figure 20:**
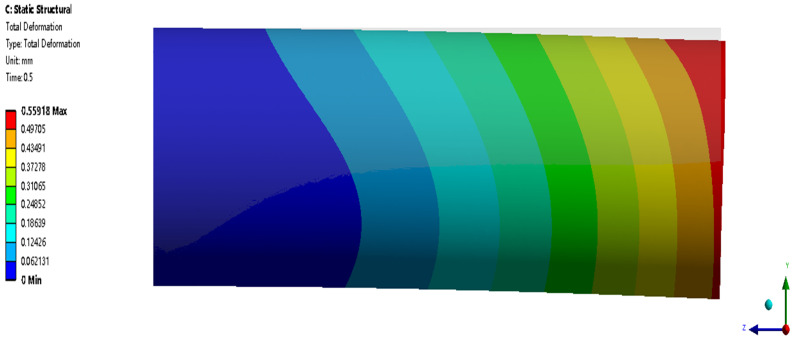
Total deformation contour of the axial actuator’s bending motion when only one chamber’s hydraulic pressure is 0.1 MPa.

**Figure 21:**
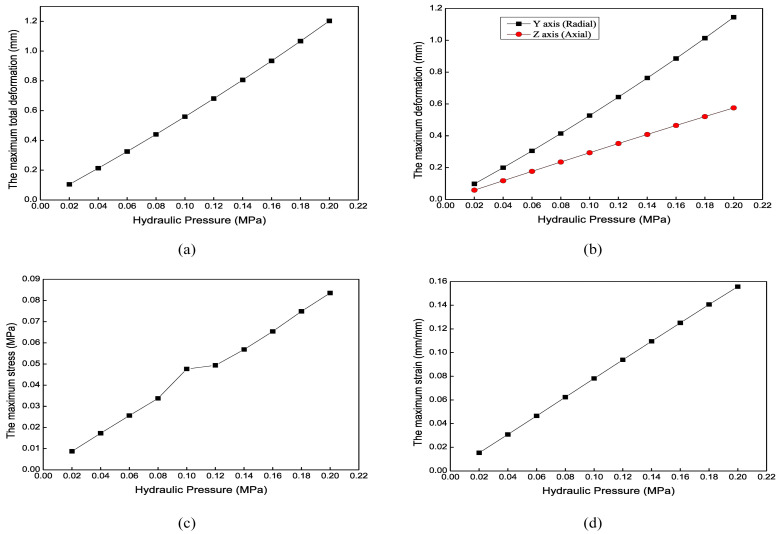
Mechanical results of the axial actuator’s bending motion under different hydraulic pressures. (**a**) The maximum total deformation. (**b**) The maximum radial and axial deformation. (**c**) The maximum stress. (**d**) The maximum strain.

In this analysis, only one chamber is filled with normal saline, and the other two are not. Adjusting the hydraulic pressure of the three chambers allows the robot to bend in all directions, making it pass through the blood vessel bifurcation more flexibly, thus achieving the bending motion. Figure 20 shows the total deformation contour of the axial actuator’s bending motion when only one chamber’s hydraulic pressure is 0.1 MPa. Figure 21 shows the mechanical results of the axial actuator bending. The analysis time is set to 0.5 s to reduce calculation, so the maximum total deformation is slight, 0.56 mm. Based on these simulation data and results, the axial actuator’s kinematic model can be established to control the soft robot’s tip position by adjusting the hydraulic pressure.

### 4.4. Simulation Results of the Radial Actuator

#### 4.4.1. Flow Field

**Figure 22:**
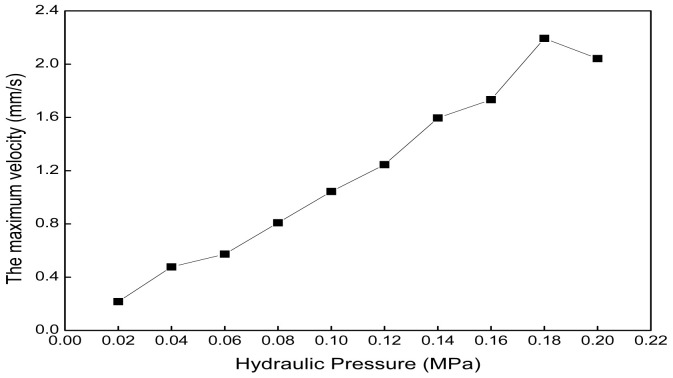
The maximum velocity of normal saline water in the radial actuator chamber under different hydraulic pressures.

Figure 22 shows that the maximum velocity of normal saline water in the radial actuator chamber increases from 0.22 mm/s to 2.04 mm/s when the hydraulic pressure increases from 0.02 MPa to 0.2 MPa. The relationship between the maximum velocity and hydraulic pressure can be established. 

#### 4.4.2. Radial Expansion Results

**Figure 23:**
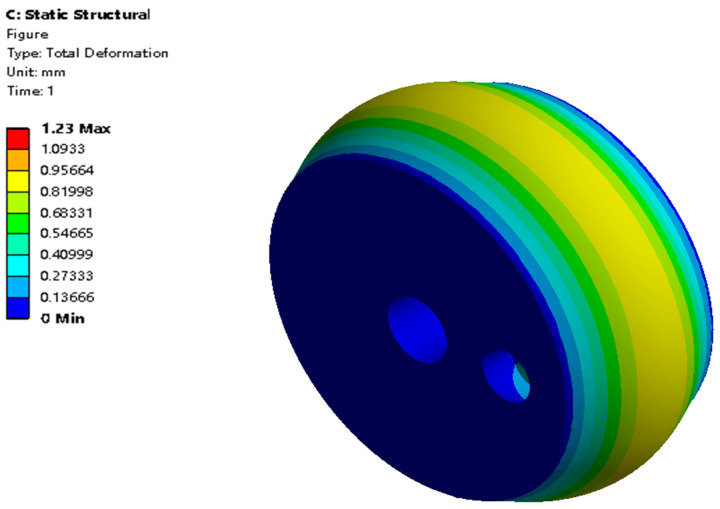
Total deformation contour of the radial actuator’s expansion motion when the chamber’s hydraulic pressure is 0.1 MPa.

**Figure 24:**
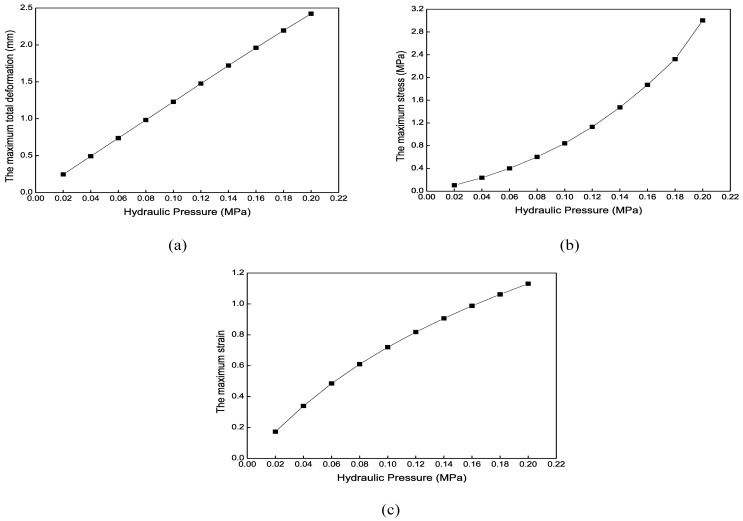
Mechanical results of the radial actuator’s expansion motion under different hydraulic pressures. (**a**) The maximum total deformation. (**b**) The maximum stress. (**c**) The maximum strain.

Figure 23 shows the maximum total deformation contour of the radial actuator’s expansion motion when the chamber’s hydraulic pressure is 0.1 MPa. The radial actuator is supported on the blood vessel through this deformation. Due to the axial constraint, the total deformation differs little from the radial deformation. Figure 24 shows that the maximum total deformation increases from 0.25 mm to 2.42 mm when the hydraulic pressure increases from 0.02 MPa to 0.2 MPa. The relationship between the maximum stress or strain and the hydraulic pressure is nonlinear.

## 5. Conclusions

A three-actuator bionic soft robot is developed based on the earthworm’s locomotion to open CTO lesions in coronary or peripheral artery vessels. The influence of the structure parameters on actuator deformation can guide the design of the soft actuators and significantly improve design efficiency. Finite element analysis is performed with the bidirectional fluid-structure coupling method to establish the kinematic model. The simulation results help us tune the actuator deformation to realize the motion control of the soft robot by changing the hydraulic pressure of the axial actuator chamber.

In the future, we need to design a better robot structure that can mimic the locomotion of earthworms, establish a more accurate kinematic model, and optimize the simpler simulation flow to obtain more accurate results with less computation. We need to present a more detailed model or an experimental prototype under ambient conditions corresponding to a real blood vessel for evaluating the feasibility of the proposed soft robot. It has good prospects for application in blood vessel medicine and other areas, e.g., fluids in small-diameter tubes.

## 6. Patents

The device presented in this article is protected by a Chinese invention patent.

## Figures and Tables

**Table 1:** Correspondence between the earthworm’s locomotion and actuator actions.

Type	Action	Radial	Axial
Circular muscles	Contraction	Shrink	Extend
Circular muscles	Relaxation	Expand	Shorten
Longitudinal muscles	Contraction	Expand	Shorten
Longitudinal muscles	Relaxation	Shrink	Extend
Radial actuators	Pumping in	Expand	Invariant
Radial actuators	Pumping out	Shrink	Invariant
Axial actuators	Pumping in	Shrink	Extend
Axial actuators	Pumping out	Expand	Shorten

**Table 2:** The material property parameters.

Material Number	$C_{1}$ (MPa)	$C_{2}$ (MPa)
1	0.1	0.02
2	0.11	0.02
3	0.12	0.02
4	0.11488	0.001232
5	0.096	0.0095

## Data Availability

The original contributions presented in the study are included in the article, further inquiries can be directed to the corresponding authors.

## Abbreviations

The following abbreviations are used in this manuscript:

CTO	chronic total occlusion
FSI	fluid–structure interaction
TIMI	thrombosis in myocardial infarction
PCI	percutaneous coronary intervention
CAD	coronary artery disease
PAD	peripheral artery disease
SFA	soft fluidic actuator
SMA	shape memory alloy
EAP	electroactive polymer
PCC	piecewise constant curvature
